# Prevalence of *Giardia duodenalis* genetic assemblages isolated from dogs and cats in Poland

**DOI:** 10.2478/jvetres-2025-0043

**Published:** 2025-08-28

**Authors:** Dawid Jańczak, Jakub Olszewski, Aleksandra Kornelia Maj, Piotr Górecki, Magdalena Nowak, Olga Szaluś-Jordanow

**Affiliations:** 1 Department of Infectious and Invasive Diseases and Veterinary Administration, Institute of Veterinary Medicine, Faculty of Biological and Veterinary Sciences, Nicolaus Copernicus University, 87-100 Toruń, Poland; 2 Animallab Veterinary Laboratory, 03-340 Warsaw, Poland; 3 Department of Food Hygiene and Public Health Protection, Institute of Veterinary Medicine, Warsaw University of Life Sciences-SGGW, 02-787 Warsaw, Poland; 4 Department of Small Animal Diseases with Clinic, Institute of Veterinary Medicine, Warsaw University of Life Sciences-SGGW, 02-776 Warsaw, Poland

**Keywords:** *β-giardin* locus, flagellate, genetic assemblage, protozoan parasite, zinc flotation

## Abstract

**Introduction:**

*Giardia duodenalis* (syn. *G. intestinalis* and *G. lamblia*) is a protozoan intestinal parasite that infects various vertebrates. Genetic analyses of *G. duodenalis* have identified eight genetic assemblages (groups), designated A–H. This study aimed to determine the prevalence of *G. duodenalis* in household dogs and cats in Poland, analyse isolates by assemblage and relate assemblage results to zoonotic potential.

**Material and Methods:**

From January to February 2024, 1,937 faecal samples from dogs and 1,077 samples from cats were examined microscopically for *G. duodenalis* cysts using the zinc flotation method. Positive samples were retained for further molecular tests. Genetic analysis was performed on the basis of the *β-giardin* locus which is 511 base pairs long. The obtained sequences were compared with reference sequences in the GenBank database.

**Results:**

*Giardia duodenalis* cysts were detected in 11.3% (219/1,937) of dogs and 7.06% (76/1,077) of cats using the zinc flotation method. Assemblages B, C, D and F were identified in 11, 77, 126 and 5 dogs, respectively. All isolates from cats were identified as assemblage F.

**Conclusion:**

The high rate of infection in pets increases the risk of transmission of zoonotic *Giardia* genetic assemblages to humans.

## Introduction

*Giardia duodenalis* (also known as *G. intestinalis* and *G. lamblia*) is a species of intestinal flagellate with worldwide distribution that infects a wide range of mammalian species, including humans, pets, livestock and wild animals (16). The protozoan *G. duodenalis* has been divided into eight distinct genotypes or assemblages labelled from A to H (46). Infections in humans are mainly caused by assemblages A (sub-assemblage AII) and B (sub-assemblage BIV); however, infections with the more typically animal-associated assemblages C, D, E and F have also been reported (37, 39, 42). Infection occurs through ingesting food or water contaminated with *G. duodenalis* cysts. Direct transmission from person to person and animal to animal *via* the faecal–oral route also occurs (12). The most frequently described symptoms of giardiasis include diarrhoea, abdominal pain, flatulence, fatty stools, weight loss and malabsorption (43). In 1979 the World Health Organization (WHO) listed *G. duodenalis* as an important zoonotic parasite (45). Moreover, along with *Cryptosporidium* genus members, *G. duodenalis* was included in the WHO Neglected Diseases Initiative in 2004 as an agent of a parasitic disease that impairs development and socio-economic improvement (38).

These flagellates are common intestinal parasites in dogs and cats. Their prevalence in European countries varies from 0.4 to 55.2% among dogs and from 1.23 to 23.08% in cats. A factor in the variation in observed prevalence is the diagnostic method employed (microscopic examination, PCR, ELISA or indirect fluorescent antibody techniques), and factors in the variation in actual prevalence are the age and origin of the animals, population type, and the presence or absence of symptoms (4, 5, 6, 17, 20, 22, 31, 32, 35, 40, 41, 48) (Table 1).

**Table 1.** Prevalence of *Giardia duodenalis* in dogs and cats in different regions of Poland between 2005 and 2021

Location in Poland	Clinical signs	Age	Origin	Detection method	Total	Positive	%	Assemblages	Reference
Dogs									
Lublin	asymptomatic	nd	city & rural comp.	ELISA	86	46	53.5	-	(17)
Warsaw	asymptomatic	nd	companion	IFA	256	47	18.4	-	(35)
Warsaw	nd	nd	nd	microscopy	350	18	5.1	A–I, C, D	(48)
Unknown	asymptomatic	> 1y	sled dogs	IFA	108	31	28.7	-	(4)
West-central	nd	nd	comp. & shelter	microscopy, PCR	148	3	2.0	C, D	(41)
Wrocław	symptomatic	3m–10y	companion	POC lateral flow coproantigen ELISA, PCR	128	27	21.1	B, C, D	(32)
Masovian Voivodeship and Lesser Poland Voivodeship	nd	nd	companion	microscopy	207	37	17.8	-	(5)
Warsaw	mixed	>1y	shelter	microscopy, PCR	74	20	27.0	C, D, F	(40)
Masovian Voivodeship	nd	3w–16y	nd	microscopy	3613	299	8.3	-	(6)
Pomeranian Voivodeship, Greater Poland Voivodeship, Lower Silesian Voivodeship, Opole Voivodship, Silesian Voivodeship, Lodz Voivodeship, Holy Cross Voivodeship, Lesser Poland Voivodeship and Subcarpathian Voivodeship	mixed	>2m–11y	companion	PCR	217	13	6.0	C, D	(31)
Cats									
Warsaw	asymptomatic	nd	city comp.	IFA	81	10	12.3	-	(35)
Warsaw	nd	nd	companion	IFA, PCR	160	6	3.8	A, B, D	(20)
Wrocław	symptomatic	3m–10y	companion	POC lateral flow coproantigen ELISA, PCR	33	5	15.2	A, F	(32)
nd	mixed	>1y	companion & stray	PCR	63	3	4.8	F	(22)
Warsaw	mixed	>1y	shelter	microscopy, PCR	13	6	46.2	D, F	(40)
Masovian Voivodeship	nd	3w–16y	nd	microscopy	1612	93	5.8	-	(6)
Pomeranian Voivodeship, Greater Poland Voivodeship, Lower Silesian Voivodeship, Opole Voivodeship, Silesian Voivodeship, Lodz Voivodeship, Holy Cross Voivodeship, Lesser Poland Voivodeship and Subcarpathian Voivodeship	mixed	>2m–11y	companion	PCR	76	3	3.9	A, F	(31)

1 nd – no available data; IFA – indirect fluorescent antibody; POC – point-of-care; w – weeks; m – months; y – years

In symptomatic dogs from seven European countries (Belgium, Estonia, France, Germany, Italy, the Netherlands and the United Kingdom), the overall percentage of positive samples was 24.8%. The highest prevalence rates were reported in Belgium (28.47%) and France (27.53%). Similar findings were observed in symptomatic cats, with the highest prevalence reported in Belgium (26.32%) and Germany (24.59%). The overall percentage of positive samples among cats was 20.3% (14). In Poland the prevalence of *G. duodenalis* in dogs ranged from 2.0 to 53.5% and in cats from 3.8 to 46.2%. The assemblages of *G. duodenalis* identified in dogs are A, B, C, D and F while assemblages A, B, D and F have been confirmed in cats (4, 8, 17, 20, 22, 31, 32, 35, 39, 40, 41, 46, 48).

This study aimed to determine the prevalence of *G. duodenalis* in companion dogs and cats in Poland, analyse isolates by assemblage and relate assemblage results to zoonotic potential.

## Material and Methods

### Sampling

Faecal samples of dogs (n = 1,937) and cats (n = 1,077) were examined microscopically between January and February 2024 in two commercial veterinary laboratories based in Warsaw and Łódź, Poland. The samples were collected from dogs and cats that were patients of veterinary clinics across the country as part of routine antiparasitic prophylaxis. The patients were assigned to <1-year-old and >1-year-old groups. The stool samples were registered in the laboratory system, which enabled them to be labelled with sex, age and origin voivodeship data. Additionally, registering the examined animals avoided repeated control tests for the same dogs and cats. This prevented an artefactual increase in the number of examined animals in cases of ineffective treatment.

### Microscopic examination

**Fig. 1.**
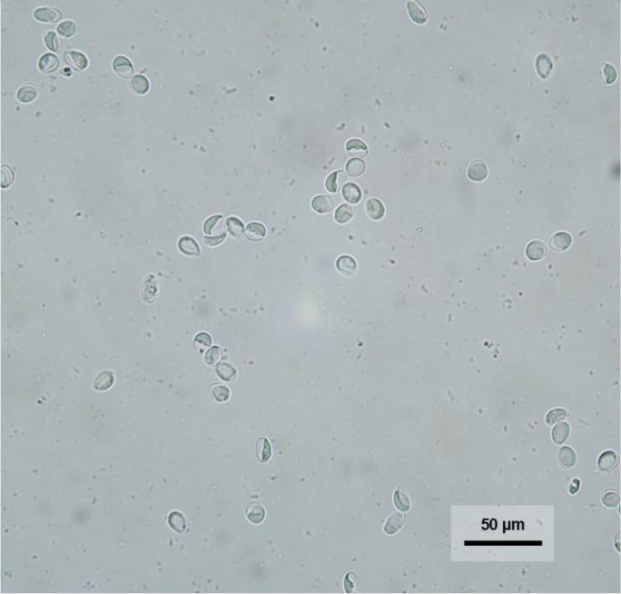
*Giardia duodenalis* cysts in Polish canine faecal samples visible in a microscopic preparation obtained by the zinc flotation method

To detect *G. duodenalis* cysts, the zinc flotation method was employed with a ZnSO_4_ solution at specific gravity of 1.31 g/cm^3^. Cysts detected on the cover slip were examined under a microscope, rinsed with distilled water and centrifuged, and the resulting precipitate was resuspended in 70% ethanol for further analysis (Fig. 1). Only positive samples were retained for molecular analysis.

### DNA isolation

Using the Sherlock AX Kit, DNA was extracted from ethanol-fixed cysts (A&A Biotechnology, Gdańsk, Poland). Prior to extraction, cysts were suspended in 300 μL of distilled water. The extracted DNA was eluted in 200 μL of the kit Tris Buffer at 10 mM and pH 8.5. The DNA samples were stored at -20°C for molecular analysis.

### Nested PCR

A nested PCR protocol was employed to amplify the β-giardin gene. In the first PCR cycle, a 753-base-pair (bp) fragment was amplified using primers G7 and G759 (9). In the second (nested) PCR, a 511-bp fragment was amplified following a previously described protocol (23) with a modification consisting of a 1 : 100 dilution of the first-round PCR products with deionised water prior to the nested PCR.

The amplicons were analysed on 2% agarose gel and the 511-bp PCR products were sequenced in both directions using the Sanger dideoxy method by an external commercial provider (DNA Sequencing and Synthesis Laboratory – Oligo IBB, Warsaw, Poland). All PCR and nested-PCR reactions were performed in a MultiGene optiMAX thermocycler (Labnet International, Taoyuan, Taiwan).

### Sequencing and analysis

**Fig. 2.**
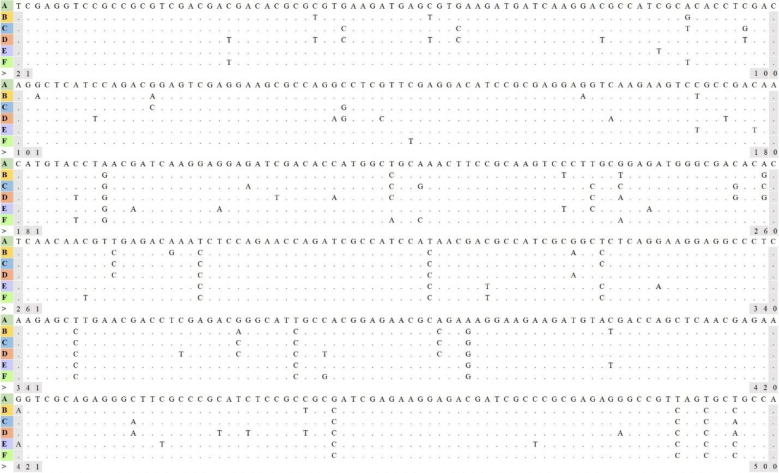
Consensus sequences of assemblages of *Giardia duodenalis* isolated from canine faecal samples in Poland

The sequencing data were analysed using Chromas v. 2.6.6 software (Technelysium, South Brisbane, QLD, Australia). To compare the obtained nucleotide sequences with the NCBI GenBank database, the basic local alignment search tool was used. The GenBank accession Nos of the reference sequence for assemblages A, B, C, D, E and F were: MG924451.1, MK982544.1, MN270296.1, MN044604.1, MT108433.1 and ON088501.1, respectively. After database matching, the *Giardia* species and assemblages were identified by comparing the consensus sequences with reference sequences using MEGA v. 7 software (21) (Fig. 2). The comparison was made using the neighbour-joining method based on nucleotide sequences of the *β-giardin* locus and the Kimura two-parameter model. Sequences of *G. psittaci* (GenBank accession No. AB714977.1), *G. agilis* (MF185954.1) and *G. muris* (MT713338.1) were used as outgroups.

### Statistical analysis

Statistical analysis was performed using the chi-squared test to assess the significance of differences in the prevalence of *G. duodenalis* in dogs and cats across age groups (<1 year old or >1 year old) and between sexes.

## Results

### Microscopic examination

**Fig. 3.**
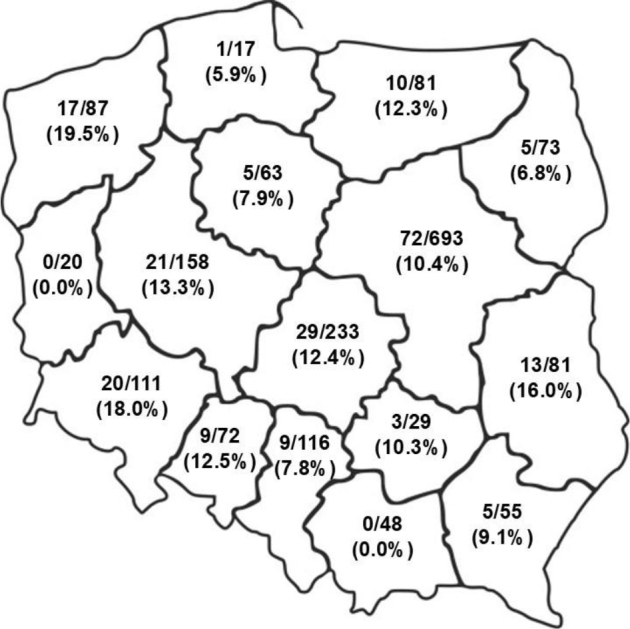
Prevalence of *Giardia duodenalis* in canine faecal samples from different voivodeships in Poland

**Fig. 4.**
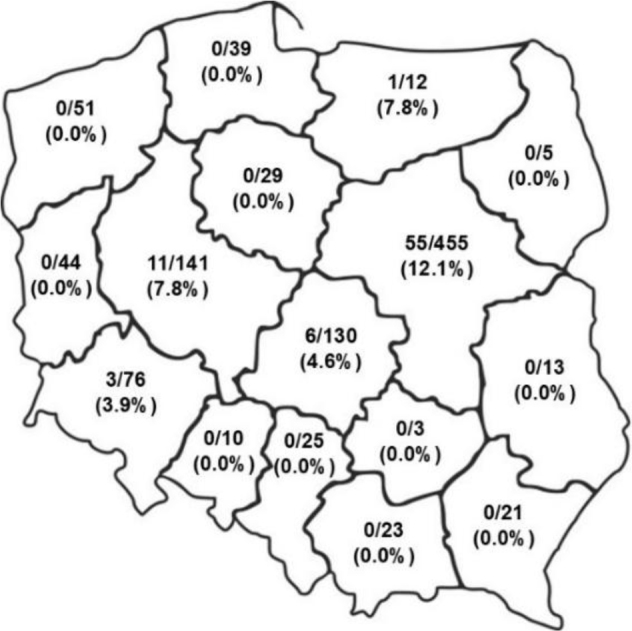
Prevalence of *Giardia duodenalis* in feline faecal samples from different voivodeships in Poland

*Giardia duodenalis* cysts were detected in 11.3% of dogs (219/1,937) and 7.1% of cats (76/1,077). The highest canine prevalence was observed in dogs younger than one year, with 16.9 % (71/419) testing positive, and the highest feline prevalence was noted in cats younger than one year, with 19.8% (43/217) testing positive. The by-voivodeship prevalence of *G. duodenalis* in dogs and cats in Poland is presented in Figs 3 and 4.

### Molecular identification

**Fig. 5.**
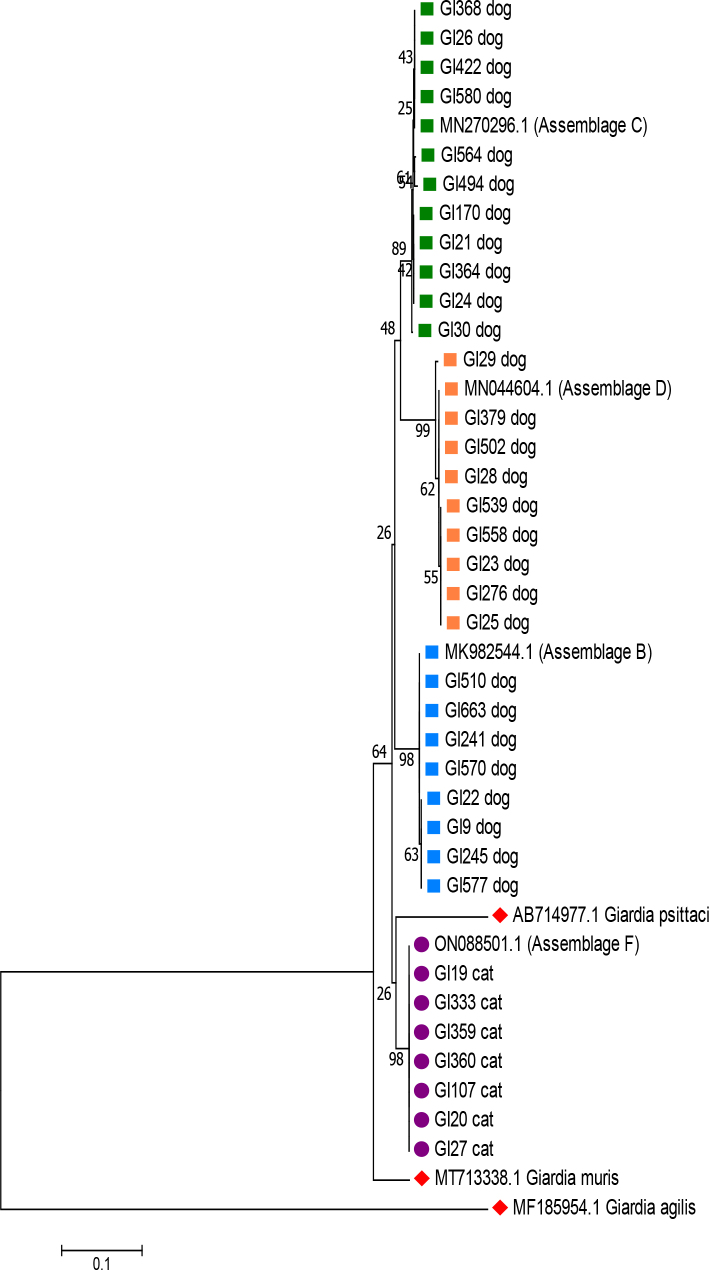
Phylogenetic relationships of selected *Giardia duodenalis* assemblages (GI) isolated from canine and feline faecal samples in Poland. Green squares – assemblage C isolates and their GenBank reference sequence; orange squares – assemblage D isolates and reference sequence; blue squares – assemblage B isolates and reference sequence; purple circles – assemblage F isolates and reference sequence; mitred red squares – outgroup reference sequences

Among the 219 *G. duodenalis* isolates obtained from dogs, those of assemblage D were the most prevalent and comprised a 57.53% proportion (126/219). Assemblage C was the next largest proportion at 35.16% (77/219). Assemblage B was detected in 11 dogs’ samples, and 5 isolates were found to be 100% homologous with assemblage F. No co-infections with more than one assemblage were identified. All *G. duodenalis* isolates obtained from cats were identified as assemblage F (Table 2). The phylogenetic relationships between *G. duodenalis* assemblages isolated from dogs and cats are presented in Fig. 5.

**Table 2.** Prevalence of *Giardia duodenalis* gene assemblages in dogs and cats by age group

	Dogs (n =1,937)		Cats (n =1,077)	
Age group	<1y	>1y	<1y	>1y
Number of animals (n)	491	1,446	217	860
Giardia-positive (%)	71 (14.5)	148 (10.2)	43 (19.8)	33 (3.8)
Assemblages				
A	0	0	0	0
B	11	0	0	0
C	19	58	0	0
D	41	85	0	0
E	0	0	0	0
F	0	5	43	33

1 y – year

### Statistical analysis

The chi-squared test revealed a significant difference in the prevalence of *G. duodenalis* between dogs and cats. Dogs were significantly more frequently infected with the parasite than cats (χ^2^(1) = 14.51, P-value < 0.001). No significant difference was observed between the group of dogs aged under one year and the older group in carriage of infection with *G. duodenalis* (χ^2^(1) = 0.55, P-value = 0.457). However, analysis of these age groups in cats revealed a significant difference: cats younger than one year were infected more frequently (χ^2^(1) = 65.04, P-value < 0.001).

The analysis of the association between sex and *G. duodenalis* prevalence in dogs revealed that 141 of 1,134 females (12.4%) and 78 of 803 males (9.7%) were infected. Although the prevalence was higher among females, the difference was not statistically significant (χ^2^(1) = 3.20, p-value = 0.073). In contrast, a statistically significant association was observed in cats, where 59 of 667 females (8.8%) and 18 of 410 males (4.4%) tested positive (χ^2^(1) = 6.94, P-value = 0.008), indicating a potential relationship between sex and infection status in this species.

## Discussion

Several studies have reported surprisingly high prevalence rates of *G. duodenalis* in animals. For instance, in Europe its prevalence among shelter dogs was found to be 55.2% (30) and 34.4% (14), and among companion dogs 27.17% (47). In Poland, a study conducted in the Lublin region in 2005 revealed that 53.5% of companion dogs without any gastrointestinal disturbances living in urban and rural areas were infected with *G. duodenalis* (17). Research from Japan indicated that 23.4% of asymptomatic puppies in pet shops had giardiasis (18). Additionally, in the same country, a prevalence rate of 39.1% was observed in companion cats without visible symptoms of *G. duodenalis* infection (19). In Poland, the highest prevalence among cats (46.2%) was reported in shelter animals (40), while the lowest (3.8%) was recorded in companion cats (20). The prevalence depends on several factors such as age and origin, and the observed prevalence depends on the method used for parasite detection. Age is one of the most critical factors affecting the incidence of giardiasis. For example, Itoh *et al*. (19) found that the infection rate in kittens under six months of age was significantly higher (49%) than that of cats aged over six years (32%). In Romania, age up to six months was identified as a risk factor for *G. duodenalis* infection in cats: the prevalence in the youngest was 30.6% *vs* 24.6% in older animals (26). Similarly, a study in Italy reported a prevalence of 32.8% in cats younger than one year compared to 22.7% in older cats. Interestingly, this study also found that lone companion cats had a higher infection rate (35.5%) than cats in groups within the same household (22.2%) (47). In our study, the prevalence of *G. duodenalis* in cats under one year of age was 19.8%, and in older cats was 3.8%. Among dogs, the prevalence was also higher in younger than in older dogs at 14.5% *vs* 10.2%. Similar findings were reported by Piekara-Stępińska *et al*. (31), who noted a higher frequency of infection in dogs under one year of age. Furthermore, a comprehensive analysis conducted across seven European countries in 2005 and 2006 demonstrated significantly higher infection rates in young animals, finding a 42.9% rate in dogs and one of 26.1% in cats younger than six months (14).

Indoor cats are generally less exposed to infections, including infections with intestinal parasites. The prevalence of infection among indoor, indoor/outdoor and outdoor cats was reported to be 33%, 45% and 53%, respectively (19). Animal homelessness also appears to be a significant factor influencing infection rates. According to Kváč *et al*. (22) 30.3% of infected cats were stray animals compared to 19.4% of companion cats.

Counter to these indications of factorial significance for the circumstances in which a cat lives, Mircean *et al*. (26) found no significant differences associated with a cat’s lifestyle, also finding none related to sex, breed, co-infections with other parasites, anthelmintic treatments, type of food or season. However, in Poland Bartosik *et al*. (5, 6) identified a significant relationship between season and *G. duodenalis* infections in dogs, infections being more prevalent in autumn and winter than in spring or summer. In contrast, no significant association was found in cats between infection status and either sex or season (6). Our study did not allow for comparison of prevalence across seasons, because the sampling period was restricted to two winter months (January and February), which was a limitation. In a study conducted on cats in Daejeon city, South Korea, sex was the only significant factor, with females being more frequently infected with *G. duodenalis* than males. Other variables such as age and the presence of diarrhoea were not statistically significant (24). In our study, we observed a similar trend in cats, where females were significantly more frequently infected than males. However, this finding may be influenced by regional differences, as described by Epe *et al*. (14).

Close physical contact between pet owners and their dogs or cats is a potential route for the transmission of zoonotic parasites and other pathogens. As described by Overgaauw *et al*. (27), 50% of pet owners allow their pets to lick their faces, and 18% of dogs and 30% of cats sleep in the owner’s bed. Additionally, 45% of cats are permitted to jump onto the kitchen sink, which could further facilitate the spread of pathogens. Water, food and soil contaminated with *G. duodenalis* cysts are recognised sources of infection for both humans and animals. *Giardia duodenalis* is one of the most commonly identified pathogens in waterborne disease outbreaks (12). The parasite has also been detected in soil samples, as exemplified by the 4.5% (28/625) positivity of samples from 67 public parks in Spain (11) and 16% (8/50) positivity of soil samples from the Andean region in the central western part of Colombia (33). Given that 39% of dog owners reportedly never pick up their dog’s faeces (27), the presence of *G. duodenalis* in soil is not surprising. These findings highlight the importance of responsible pet ownership and environmental hygiene in reducing the risk of zoonotic transmission.

Accurate diagnosis and genetic characterisation are therefore essential for understanding the zoonotic potential of *G. duodenalis* infections in companion animals. The sensitivity of a PCR can vary depending on the target gene locus and the specific method employed. Compared with the traditional PCR, the nested PCR significantly enhances the sensitivity and specificity of PCR amplification (10). Tests with PCR have also been shown to be more sensitive and specific than microscopic methods. A comparison between microscopy and PCR targeting the *glutamate dehydrogenase* (*gdh*) locus for diagnosis of *G. duodenalis* demonstrated that whereas microscopy yielded 64.4% sensitivity and 86.6% specificity, molecular techniques achieved 100% sensitivity and 100% specificity (13). However, Pallant *et al*. (28) recorded the lowest amplification success for *gdh* locus and the highest for the 18S rDNA gene, with similar results observed for faecal samples from both dogs and cats. A comparable, although not statistically significant, trend was noted for the *β-giardin* PCR, with amplification failure occurring in 65% (39/60) of feline faeces samples and 53% (69/130) of canine faeces samples.

In the present research, we employed a nested PCR procedure for a 511-bp fragment of the *β-giardin* gene (9, 23), as this locus is one of the most used for analysing *G. duodenalis* assemblages isolated from humans and animals (16, 36). However, the *β-giardin* locus is not considered an optimal choice for *G. duodenalis* in clinical samples (44). Therefore, in our study, only samples that tested positive using the zinc flotation method were subjected to the nested PCR procedure. It is important to note that not all positive samples are successfully amplified at gene loci, which previous research has proved. For example, five stool samples that tested positive using a point-of-care lateral flow coproantigen ELISA which detected *G. duodenalis* antigen were PCR negative, even after increasing the matrix volume and re-isolation of DNA (32). This discrepancy can be explained by an insufficient number of parasites in the collected stool samples (3).

A comparison of four diagnostic methods used to detect chronic, subclinical *G. duodenalis* infection in dogs revealed differences in specificity and sensitivity (34). The highest sensitivity was reported for immunofluorescent cyst detection, followed by zinc sulphate flotation (pooled samples), a point-of-care lateral flow coproantigen ELISA and a traditional plate-based coproantigen ELISA, the lowest sensitivity having been observed in single-sample zinc sulphate flotation. The authors emphasised that all of the evaluated tests were capable of detecting chronic infection, even in the absence of clinical signs of giardiasis. However, in clinical practice, the use of single tests such as zinc flotation or the lateral flow coproantigen ELISA may result in false-negative findings and could lead to an incorrect exclusion of giardiasis as the cause of clinical symptoms (34).

The high importance of giardiasis as a public health problem and its significant zoonotic potential make determining the protozoal assemblages an essential part of giardiasis diagnosis in infected household pets, even if the transmission route of zoonotic or host-specific assemblages of *G. duodenalis* from animals to humans remains unestablished definitively (7). In our study, among 219 *G. duodenalis-*positive faecal samples from dogs, 11 were identified as having parasite content with genetic assemblage B, which is commonly detected in both humans and animals (9). Although it has been established that the animal-specific assemblages C, D, E and F typically transmit *via* host-specific routes, these too have occasionally caused infection in humans (37, 39, 42). All assemblages identified in the research which we undertook were potentially zoonotic, with assemblage B detected in 11 dogs appearing to be the most significant infectious agent. Moreover, the epidemiological investigation confirmed that these 11 dogs were puppies adopted from the same exact location in Warsaw, suggesting the possible circulation of a single assemblage within this origin site. Humans are primarily infected by zoonotic assemblages A and B, where sub-assemblages AII and BIV are the predominant variants identified in human cases (37). Among household animals, assemblage A was the most frequently reported in cats (20, 31, 32, 37) dogs (15, 29, 48), pet chinchillas (25) and pet ferrets (1, 2). Assemblage B is the predominant cause of infections in dogs, alongside assemblages C and D to lesser extents and occasionally F (10, 37, 46). In our study, some *G. duodenalis*-positive dogs were infected with assemblages C (19/71) and D (41/71) besides B. Similar findings were reported by Zygner *et al*. (48), who identified infections with assemblages AI (1.71%), C (1.14%) and D (6.28%) among 350 dogs from Warsaw (48). In another Polish city, Wrocław, assemblage C (18/22) was the predominant variant in dogs, while assemblages D and B were detected in two and one dog, respectively (32). Additionally, Solarczyk and Majewska (41) reported the presence of only assemblages C and D in three dogs infected with *G. duodenalis*. Assemblage F, although typically considered highly specific to feline hosts, has been shown to infect humans and dogs (39). Five of the present study’s dogs over one year of age were found to be infected with the F assemblage. Similarly, a previous study focusing on shelter animals reported that 8 of 20 *G. duodenalis*-positive dogs carried the F variant (40). Additionally, all samples obtained from cats in our study were identified as assemblage F.

## Conclusion

Our study demonstrates that *G. duodenalis* infections are common among household animals. However, the direct role of dogs and cats in the transmission of zoonotic strains to humans remains unclear. Nonetheless, because of the particular environmental contamination to which pets are more exposed than their owners, and the close physical contact between pet owners and their animals, the risk of zoonotic transmission persists. Even asymptomatic animals showing no gastrointestinal symptoms can be sources of infection. As highlighted in our research, zoonotic assemblages capable of infecting humans may circulate among animals from a single origin site.
